# Placebo effects improve sickness symptoms and drug efficacy during systemic inflammation: a randomized controlled trial in human experimental endotoxemia

**DOI:** 10.1186/s12916-025-04292-8

**Published:** 2025-08-04

**Authors:** Justine Schmidt, Johanna Reinold, Hana Rohn, Manfred Schedlowski, Harald Engler, Sigrid Elsenbruch, Sven Benson

**Affiliations:** 1 Institute for Medical Education, Center for Translational Neuro- and Behavioral Sciences (C-TNBS), University Hospital Essen, University of Duisburg-Essen, Essen, 45122 Germany; 2 Institute of Medical Psychology and Behavioral Immunobiology, C-TNBS, University Hospital Essen, University of Duisburg-Essen, Essen, 45122 Germany; 3 Department of Infectious Diseases, University Hospital Essen, University of Duisburg-Essen, Essen, 45122 Germany; 4 Department of Clinical Neuroscience, Osher Center for Integrative Medicine, Karolinska Institutet, Stockholm, 171 77 Sweden; 5 Department of Medical Psychology and Medical Sociology, Faculty of Medicine, Ruhr University Bochum, Bochum, 44901 Germany; 6 Department of Neurology, C-TNBS, University Hospital Essen, University of Duisburg-Essen, Essen, 45122 Germany

**Keywords:** Inflammation, Endotoxemia, Lipopolysaccharide, Placebo effect, Expectations, Sickness behaviour, Depression, Anxiety, Ibuprofen, Balanced placebo design

## Abstract

**Background:**

Systemic inflammation triggers a wide range of sickness symptoms, including bodily discomfort and affective symptoms, which are relevant to numerous health conditions. While extensive research in the placebo field demonstrates that positive expectations can improve symptoms, it remains unclear if interventions designed to augment positive treatment expectations can alleviate sickness symptoms in the context of immunomodulatory drug therapies.

**Methods:**

In this randomized, controlled, fully balanced 2 × 2 factorial placebo design, *N* = 124 healthy volunteers received either active ibuprofen treatment (600 mg per os) or placebo, combined with either a positive or neutral labeling of the treatment by the physician. All participants were intravenously injected with lipopolysaccharide (LPS, 0.8 ng per kg of body weight) as a translational model of inflammation-induced sickness symptoms. Primary outcomes were bodily and affective symptoms, assessed at baseline and up to 6 h after injection, along with a range of inflammatory markers.

**Results:**

Ibuprofen substantially alleviated inflammation-induced symptoms. Positive labeling also improved bodily and affective symptoms of sickness, even in placebo-treated groups. Notably, positive labeling enhanced ibuprofen’s efficacy for alleviating affective symptoms, supporting that expectations can boost the efficacy of a highly effective anti-inflammatory treatment. However, labeling did not influence changes in physiological markers of inflammation, suggesting that the effects of expectations primarily act through mechanisms distinct from direct modulation of peripheral immune responses.

**Conclusions:**

Placebo mechanisms engaged by physician communication can independently alleviate inflammation-induced symptom burden and enhance the efficacy of an anti-inflammatory medication. Results underscore the critical role of healthcare provider communication and pave the way for improved treatment strategies for conditions characterized by inflammation-driven symptoms.

**Trial registration:**

DRKS00023088, registration website German Clinical Trials Register (date registered: 10/22/2020).

**Supplementary Information:**

The online version contains supplementary material available at 10.1186/s12916-025-04292-8.

## Background

Placebo and nocebo effects shape health outcomes in the context of immunotherapies and vaccinations [1, 2], with broad implications for symptom and side effect experiences, therapy adherence, and health-related quality of life [3]. The impact of expectations on inflammation-induced symptoms became particularly evident during the COVID-19 pandemic, notably in the context of nocebo responses observed in vaccination trials (e.g., [1]). While extensive research in the placebo field has demonstrated that positive expectations can alleviate various bodily and psychological symptoms [4, 5] that also characterize states of inflammation-induced sickness [6–9], translational placebo research on sickness symptoms remains limited. As a key driver of placebo effects, physician–patient communication shapes treatment expectations, with significant benefits for treatment outcomes [4, 10]. Importantly, first evidence is emerging that interventions boosting positive treatment expectations such as a positive labeling of treatments may also enhance drug efficacy (e.g., [11–14], paving the way for personalized and patient-centered treatment regimens [10]. Given the transdiagnostic significance of inflammation-induced sickness symptoms in chronic inflammatory conditions and psychiatric disorders [6, 7], research targeting placebo mechanisms within the context of systemic inflammation and anti-inflammatory therapies is warranted.

Human experimental endotoxemia serves as a robust translational model for inflammation-induced sickness symptoms, allowing to test both pharmacological and psychological interventions [8]. Administering low-dose purified bacterial endotoxin (e.g., lipopolysaccharide, LPS) to healthy individuals reliably induces systemic immune activation characterized by transient increases in inflammatory mediators such as pro-inflammatory cytokines and prostaglandins. This activation results in bodily discomfort and malaise, as well as psychological symptoms, including depressed mood and anxiety (reviewed in [8, 15]). Initial correlative studies suggest that expectations influence LPS-induced sickness symptoms [16–18] supporting the putative relevance of placebo and nocebo effects. However, it remains unknown whether an intervention designed to augment positive treatment expectations can significantly improve anti-inflammatory drug treatment efficacy.

In this randomized controlled study, we implemented a fully balanced 2 × 2 factorial design to examine the main and interaction effects of augmented positive treatment expectations and anti-inflammatory medication. In one of the largest human endotoxin studies to date, *N* = 124 healthy volunteers received an intravenous injection of LPS (0.8 ng per kg of body weight). We chose the translational LPS model because it allows us to induce clinically relevant symptoms and to assess symptom modulation under strictly standardized experimental conditions. LPS injection was preceded by randomized and double-blind administration of either an active anti-inflammatory drug, i.e., the nonspecific cyclooxygenase inhibitor ibuprofen, or an identically looking inert placebo capsule. Ibuprofen or placebo was randomly paired with either positive or neutral treatment-related information, i.e., different labeling of the treatment, modeling variability in provider-patient communication as one major source of placebo effects in routine patient care.

As primary outcomes, we measured self-reported bodily symptoms, representing the typical burden of sickness, and state levels of depression and anxiety, as indicators of affective states relevant to mental health, along with fatigue as a common symptom of inflammatory states. Symptoms were repeatedly assessed with validated questionnaires to capture the dynamic changes in LPS-induced symptoms across the study day. Secondary outcomes included physiological measures, including immune, vital, and neuroendocrine parameters. We hypothesized that positive labeling of treatment reduces LPS-induced bodily and affective sickness symptoms (main effect of labeling) and enhances the efficacy of ibuprofen (labeling × medication interaction). Secondary analyses were conducted to follow up significant labeling effects within specific subgroups of the balanced placebo design. To this end, effects of positive and neutral labeling were separately tested within the medication arm, which clarifies labeling effects within drug-treated individuals. Labeling effects were similarly tested within the placebo arm, providing information on labeling effects that reflect psychological influences on outcomes as in any typical setting of placebo studies, i.e., with inert treatment. Additionally, we explored labeling and medication effects on physiological measures, expanding on existing placebo research involving conditioning of the immune response [2].

## Methods

### Aim, design and setting of the study

In this randomized, placebo-controlled, double-blind experimental study, all *N* = 124 participants received an intravenous injection of LPS (0.8 ng per kg of body weight, details on endotoxemia below) to induce acute systemic inflammation along with sickness symptoms, following established protocols [19, 20].

**Fig. 1:**
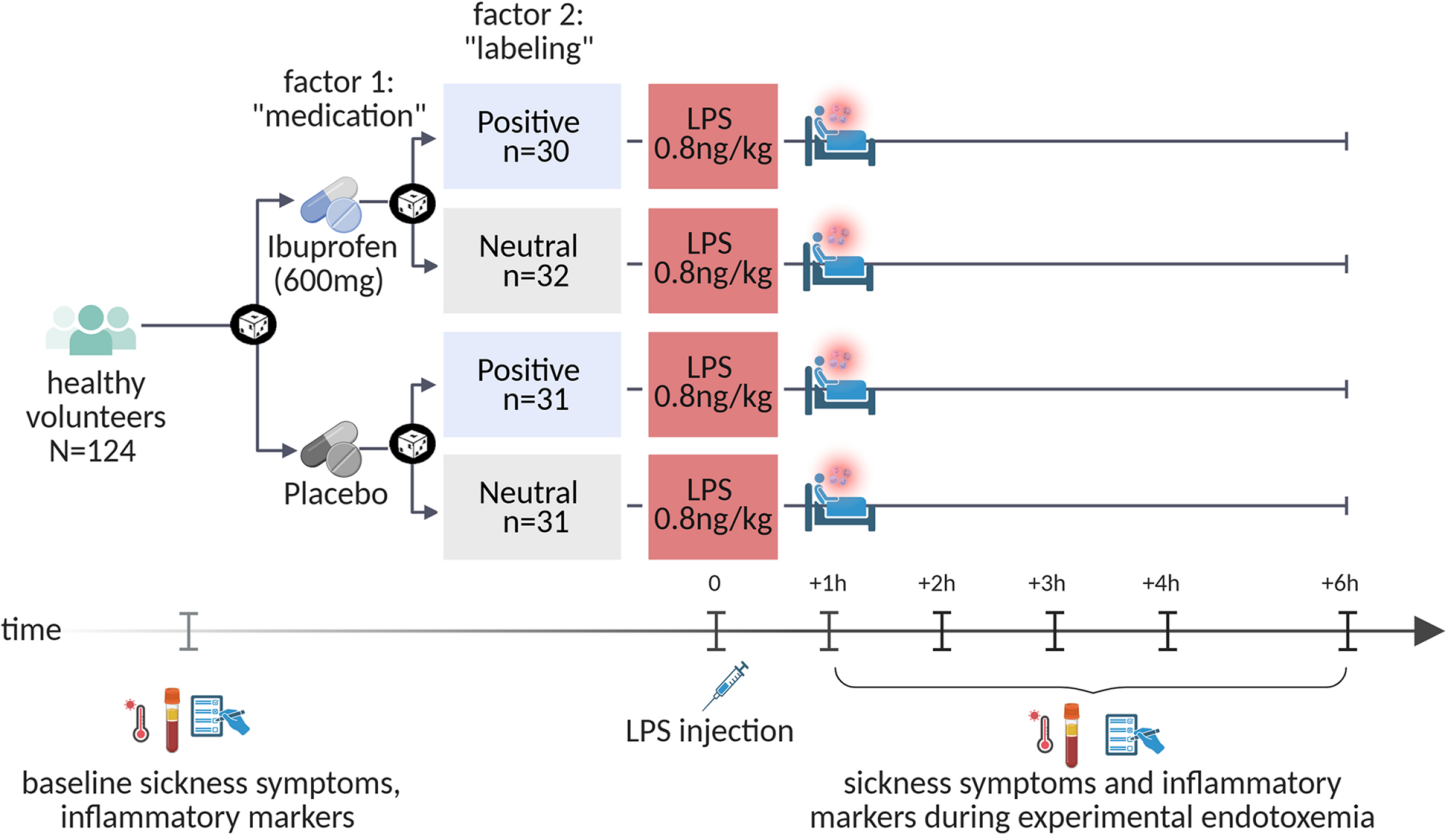
Study design*.* All volunteers were intravenously injected with endotoxin (LPS: 0.8 ng/kg of body weight) to induce acute systemic inflammation. In a 2 × 2 factorial between-group design, volunteers were randomized to receive ibuprofen or placebo (factor 1 “medication”) prior to injection, randomly combined with positive or neutral verbal treatment information delivered by the study physician (factor 2 “labeling”). Outcome measures (i.e., bodily and affective sickness symptoms, inflammatory, neuroendocrine and vital parameters) were assessed prior to LPS injection (i.e., baseline) as well as 1, 2, 3, 4, and 6 h after injection. Created with BioRender.com

The study aim was to examine the main and interaction effects of positive treatment expectations and anti-inflammatory medication. To this end, we implemented a fully balanced 2 × 2 factorial study design [4] (see Fig. 1). Participants were randomized to receive a single oral dose of either 600 mg ibuprofen or an inert substance (placebo) prior to LPS administration. The administration of ibuprofen or placebo was randomly paired with standardized verbal labeling delivered by the study physician to induce either positive or neutral expectations. This resulted in four experimental groups:

1. Ibuprofen combined with positive labeling (IBU + pos)
2. Ibuprofen combined with neutral labeling (IBU + neu)
3. Placebo capsule combined with positive labeling (PLA + pos)
4. Placebo capsule combined with neutral verbal labeling (PLA + neu)

The 2 × 2 factorial, balanced placebo design allows the investigation of the effects of physician communication (main effect of labeling), anti-inflammatory medication (main effect of medication), and their interaction (labeling × medication interaction) on outcomes.

The study was conducted at the University Hospital Essen, University of Duisburg-Essen, Germany, as part of the Collaborative Research Center (CRC) 289 “Treatment Expectation”, funded by the German Research Foundation (Deutsche Forschungsgemeinschaft, DFG). The overarching goal of the CRC is to elucidate mechanisms and clinical implications of treatment expectations. This study was accomplished as part of subproject A11 (PIs: authors S.B., H.R.), which addresses expectancy effects in the context of inflammation. The work was conducted in accordance with the Declaration of Helsinki, approved by the local Institutional Ethics Review Board (19–8755-BO), and preregistered in the German Clinical Trials Register (DRKS) on October 22nd 2020 (before inclusion of the first participant on March 2nd, 2021), registration number DRKS00023088; for further details, see trial registration website: German Clinical Trials Register. The manuscript adheres to the CONSORT guidelines and BMC's editorial policies. All participants provided written informed consent and received financial compensation for their participation, which involved multiple visits before and after the study day for screening and safety purposes. Study procedures included the administration of LPS and the assessment of outcomes, such as inflammation-related markers derived from blood samples and sickness symptoms (reported herein). Additional experimental outcomes were acquired using a modified Posner task [21] for measures elucidating the influence of emotional distractors on spatial attention and algometry for pressure pain sensitivity [22], which will be reported elsewhere given our focus on patient-reported outcomes relevant to sickness symptoms herein. Further assessments were conducted as part of the CRC’s central projects, which coordinate and administrate standardized collection of additional measures across all study sites and subprojects involving human participants for merged analyses (for a preregistration, see: OSF Registries | SFB289—Central Project NeuroImaging). These data include brain imaging assessment of structural and functional brain connectivity prior to experimental study protocols; assessment of cortisol awakening response and salivary alpha amylase activity in saliva; and a comprehensive questionnaire battery. Embedded within the larger CRC approach, these measures will serve analyses across projects aiming to identify predictors of expectancy effects, along with additional psychological trait and state measures related to stress, negative affectivity, and pain, as accomplished for example herein [23]. For the purposes of sample characterization herein, we report sociodemographic characteristics and trait anxiety and depression measured with the trait version of the validated German State-Trait Anxiety Depression Inventory (STADI) [24].

### Participants

Healthy men and women aged 18–45 years were newly recruited for this study through public advertising. All volunteers received detailed information about the study and underwent a highly standardized and well-established medical examination and screening process as implemented in our previous studies (e.g., [16, 19, 20]). This process included a thorough medical history, physical examination, and repeated assessments of laboratory parameters [blood cell count, liver enzymes, renal retention parameters, coagulation factors, C-reactive protein (CRP)]. General exclusion criteria included a history and/or current signs of gastrointestinal, hepatic, renal, cardiovascular, hematologic, or respiratory disease; obesity, diabetes mellitus, or other metabolic disorders; abnormal laboratory values; regular use of medications, especially analgesics; any contraindication to ibuprofen use; evidence of depression and anxiety based on the validated Hospital Anxiety and Depression Scale (HADS); MRI-specific exclusion criteria (phobic anxiety, claustrophobia, ferromagnetic implants, etc.); pregnancy or lactation. On the study day, women were tested for pregnancy using a urine hCG test. Sex assigned at birth was collected as self-report (male, female, diverse). As the study was conducted during the COVID-19 pandemic, additional safety measures were implemented in cooperation with the Department of Infectious Diseases, University Medicine Essen. These measures ensured the protection of study participants and personnel, and met the requirements of the health authorities. They included repeated COVID-19 testing and a mandatory six-week interval between the participant’s last COVID-19 vaccination (or last symptom of a coronavirus infection) and study entry.

Note that the originally planned sample size of *N* = 168 could not be achieved due to a 9-month delay in study start caused by the COVID-19 pandemic. For the current sample size of *N* = 124, post hoc power analysis using G*Power (version 3.1.9.7) indicated a sufficient statistical power (1-*β* = 0.98) to detect small to medium-sized interaction effects (*f* = 0.15, *α* < 0.05) in the primary outcomes. All raw data are provided in Additional file S1.

### Experimental endotoxemia

Lyophilized LPS (reference standard endotoxin from *Escherichia coli* O113:H10, lot H0K354, United States Pharmacopeia, Rockville, MD, USA) was prepared for human use as previously described [19] and underwent microbial safety testing at a cGMP-certified laboratory (Labor LS, Bad Bocklet, Germany). On the study day, an intravenous catheter was placed in an antecubital forearm vein for repeated blood withdrawals and LPS injection. All volunteers were injected with a dose of 0.8 ng LPS per kg of body weight, which has been demonstrated by our and other groups [20, 25, 26] to reliably induce a systemic immune activation and sickness symptoms. Participants were instructed to refrain from strenuous physical activity for 48 h before and on the day of the study. For safety, volunteers were continuously monitored by the study physician for 6 h after LPS injection and returned to the laboratory for follow-up visits 24 h and 1 week after injection.

Notably, the study did not include a non-LPS condition, as its primary objective was to examine the effects of psychological (labeling) and pharmacological (ibuprofen) interventions on LPS-induced symptoms. The effects of LPS versus saline injections have been extensively analyzed by our and other research groups across all study outcomes, consistently demonstrating no or negligible changes in inflammatory parameters and sickness symptoms in non-LPS (saline) conditions [16, 19, 20, 25, 26].

### Randomization and masking

Randomization for ibuprofen versus placebo and positive versus neutral labeling was conducted using an online tool (www.randomizer.org) by a person not involved in the study. The study physician received a sealed envelope containing the identical-looking ibuprofen or placebo capsule (blinded) as well as an instruction regarding the treatment-related labeling. The ALIIAS software tool [27], developed and utilized within the CRC***,*** was employed to generate a pseudonym allowing sharing and integration of data into the CRC’s data cloud.

### Anti-inflammatory or inert medication

Forty-five minutes prior to the LPS injection, volunteers received either the anti-inflammatory drug ibuprofen (1 capsule, 600 mg per os) or an identically appearing but inert capsule as a placebo, administered in a randomized, double-blind manner. The choice of drug and dosage was informed by evidence that ibuprofen (i.e., 3 × 800 mg/day) effectively reduces fever, headache, and myalgia in previous endotoxemia studies utilizing significantly higher doses of LPS (i.e., 2–4 ng per kg of body weight) [28]. The timing of drug administration was selected to ensure that the pharmacological effect coincided with the LPS injection, accounting for the delayed onset of action due to the encapsulation of the ibuprofen active ingredient. Encapsulation was necessary to maintain blinding by fabrication of a capsule identical in appearance to the inert substance, as provided by the University Hospital’s central pharmacy.

### Positive or neutral labeling

**Fig. 2:**
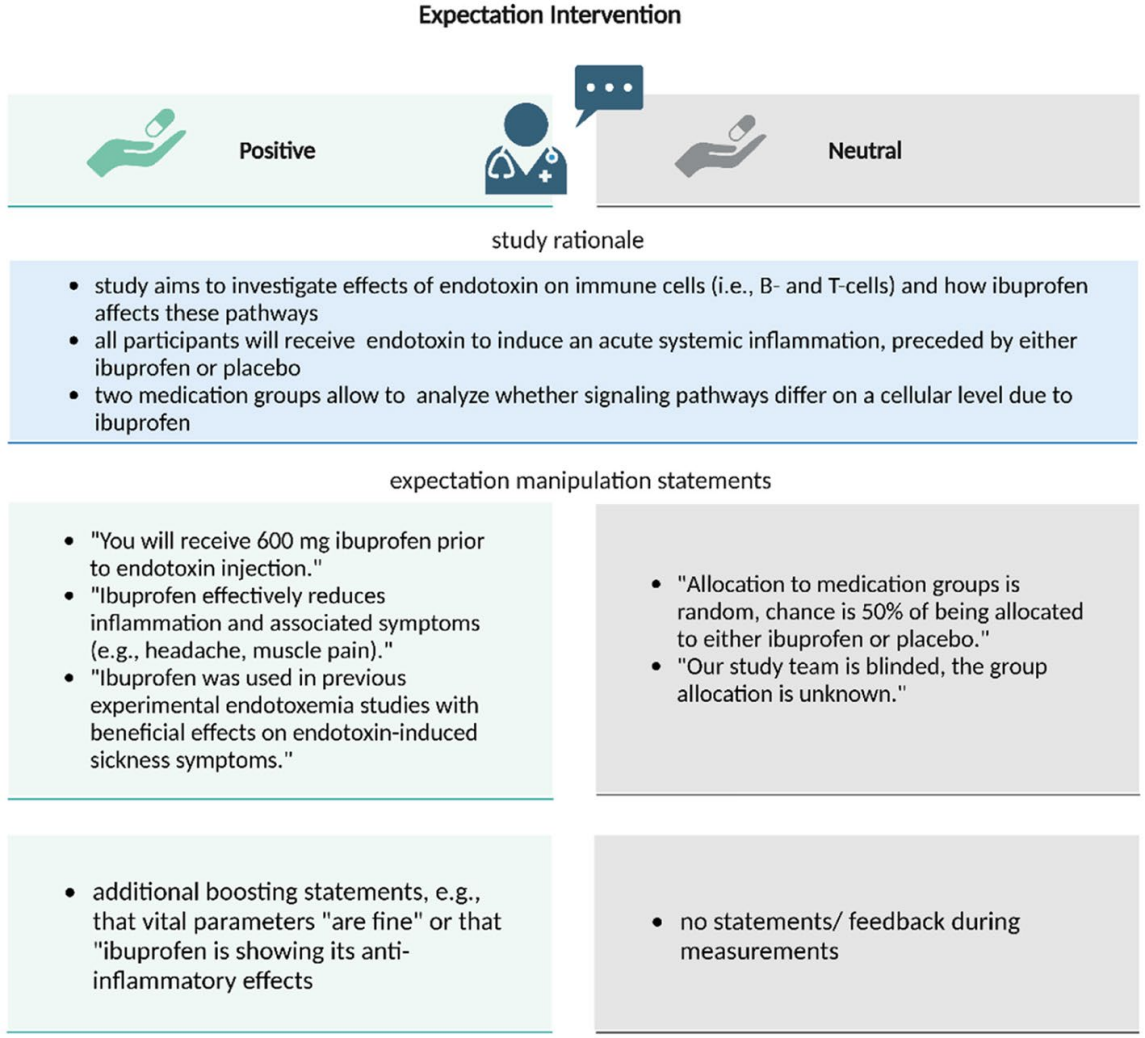
Overview of the labeling conditions: The administration of ibuprofen or placebo was randomly paired with a positive (vs. neutral as a control) labeling delivered by the study physician to augment positive treatment expectations (Positive) vs. a control condition (Neutral) based on distinct treatment information delivered by the physician. Elements of the positive labeling were repeated as booster statements. Created with BioRender.com

To augment positive treatment-related expectations, the administration of ibuprofen or placebo was randomly paired with a positive (vs. neutral as a control) labeling delivered by the study physician (see Fig. 2). In the positive labeling condition, participants were informed: “You are receiving the well-established anti-inflammatory drug ibuprofen which has been shown in previous studies to effectively improve sickness symptoms”. To reinforce this information, elements of the positive labeling were repeated by the study physician during blood sampling as booster statements (after symptom ratings to avoid directly influencing symptom reporting).

In the neutral labeling control condition, participants were informed: “You will receive either an inert substance (“sugar pill”) for control purposes or ibuprofen, with a 50%/50% chance as is usual in experimental settings”.

The positive and neutral treatment communication was scripted and delivered in a standardized manner. Adherence to the protocol was evaluated by an independent observer as previously described [13]. Similar procedures and instructions have been successfully used by our group [13].

### Outcomes

#### Sickness symptoms

Self-reported bodily and affective sickness symptoms were assessed with validated questionnaires at baseline and 1, 2, 3, 4, and 6 h after LPS injection (see study design, Fig. 1) following established procedures (e.g., [19, 20]). All questionnaires were completed digitally with the open-source application LimeSurvey (LimeSurvey GmbH, Hamburg, Germany).

The number and intensity of bodily sickness symptoms were evaluated with an adapted version of the Generic Assessment of Side Effects Scale (GASE). Briefly, volunteers rated the severity of 23 physical symptoms on a 4-point Likert scale (0 = “not present”, 1 = “mild”, 2 = “moderate”, 3 = “severe”) as previously accomplished (e.g., [19]). For the calculation of the sum score, symptoms were included only if volunteers attributed the symptoms to endotoxin administration (endotoxin-attributed total score), reflecting the severity of symptoms associated with endotoxin exposure. The resulting sum score was used for analysis, with higher scores indicating greater bodily sickness. Twenty-four hours after the LPS injection, volunteers were asked to rate their overall symptom burden retrospectively using the GASE.

For inflammation-induced changes in affective symptoms, we implemented the state version of the STADI [24]. It comprises 20 Likert-scaled items ranging from 1 (“not at all”) to 4 (“very much”). The 10-item subscale “Anxiety” comprises the affective (agitation) and cognitive (apprehension) dimensions of anxiety. The 10-item subscale “Depression” measures positive (euthymia) and depressed/negative (dysthymia) mood. The “Global score” combines the anxiety and depression subscales, with higher scores indicating more pronounced negative affectivity.

Additional measures of mood-related symptoms of sickness and fatigue, which were previously established in the context of LPS studies [19, 20, 22], were used to validate and refine the results. These measures included the State-Trait Anxiety Inventory (STAI, state version) to measure state anxiety [29]; the Multi-Dimensional Mood Questionnaire (MDBF, subscale positive mood) to assess changes in positive mood [30]; and the Karolinska Sleepiness Scale (KSS) to indicate fatigue [31, 32]. Affective and fatigue symptoms were also measured 24 h after LPS injection to ensure a return to baseline and as a safety measure. For more information, see the Additional file 2, sections S1 and S2.

#### Inflammatory, neuroendocrine and vital parameters

To document the time course and extent of LPS-induced systemic inflammation as well as the effects of the interventions, blood samples were collected before (baseline) and 1, 2, 3, 4, and 6 h after LPS injection. Blood samples were collected in ethylenediaminetetraacetic acid (EDTA) treated cubes (7.5 ml S-Monovette® EDTA, Sarstedt AG, Nürnbrecht, Germany), immediately separated by centrifugation (10 min, 4 °C, 2000 × g), and stored at −80 °C until analysis. Plasma concentrations of Tumor necrosis factor-α (TNF-α) and Interleukin (IL)−6 were measured using commercial enzyme-linked immunosorbent assays (ELISA; Quantikine® IL-6/Quantikine® high-sensitivity TNF-α ELISA; R&D Systems, Minneapolis, Minnesota) according to the manufacturer’s protocol and quantified with a Fluostar OPTIMA Microplate Reader (BMG Labtech, Offenbach, Germany). The minimum detectable dose (MDD) as an indicator of the assays’ sensitivity was 0.70 pg/mL for IL-6 and 0.022 pg/mL for TNF-α. As an indicator of hypothalamic–pituitary–adrenal (HPA)-axis activity, plasma levels of adrenocorticotropic hormone (ACTH) and cortisol were measured using commercial ELISA (IBL International, Hamburg, Germany). The MDD was 4.03 ng/mL for cortisol and 1 pg/mL for ACTH. Body temperature was measured using an auricular thermometer (GeniusTM tympanic thermometer), Covidien, Mansfield, USA) and heart rate with an automated device (Omron HBP-1300, Omron Healthcare Co. Ltd., Kyoto, Japan).

### Statistical analyses

Statistical analyses were performed with SPSS (Statistical Package for the Social Sciences), Version 29.0.2.0 (IBM Corp., IBM SPSS Statistics for Windows, Armonk, NY, USA) and G*Power (Version 3.1.9.7). Data were plotted using GraphPad Prism 9 (GraphPad Software, San Diego, CA, USA). In case of significant deviations from normal distribution (i.e., IL-6, TNF-α, ACTH, cortisol), logarithmic transformations were applied for statistical analysis. For visualization purposes, plotted results show untransformed data.

Sociodemographic and psychological variables in the experimental groups were compared using univariate analysis of variance (ANOVA) or chi^2^-tests (for dichotomous variables). For primary and secondary outcomes, repeated measures (rm) ANOVAs were computed with the within-subject factor “time” (i.e., repeated assessments of outcomes at the experimental day at 6 time points) and the between-subjects factors “medication” (ibuprofen, placebo) and “labeling” (positive, neutral). In case of violation of the assumption of sphericity, Greenhouse–Geisser correction was used, and corrected degrees of freedom (df) are reported. Significant main or interaction effects were followed up in secondary analyses: To test our directed hypotheses that positive labeling improves outcomes, positive and neutral labeling groups were separately compared within the medication and within the placebo pill conditions, respectively. For subgroup comparisons, one-tailed independent t-tests were used at single time points (with Bonferroni correction for multiple comparisons of subgroups). Primarily for visualization purposes in figures, changes in symptom scores from baseline to the peak of inflammation (i.e., 3 h post-injection) were computed as delta of peak minus baseline and compared with independent *t*-tests. Higher positive change scores indicate a greater increase in symptoms. Finally, additional correlational and regression analyses were conducted to explore whether inflammation-induced affective symptoms were related to inflammatory markers and/or to bodily symptoms (reported in Additional file 2, section S3). Alpha level was set at 0.05 unless otherwise indicated, and results are shown as mean ± standard error of the mean (SEM).

### Role of the funding source

This work was funded by the Deutsche Forschungsgemeinschaft (DFG, German Research Foundation: project-ID 422744262–TRR 289). The funder of the study had no role in study design, data collection, data analysis, or writing of the report.

## Results

**Fig. 3:**
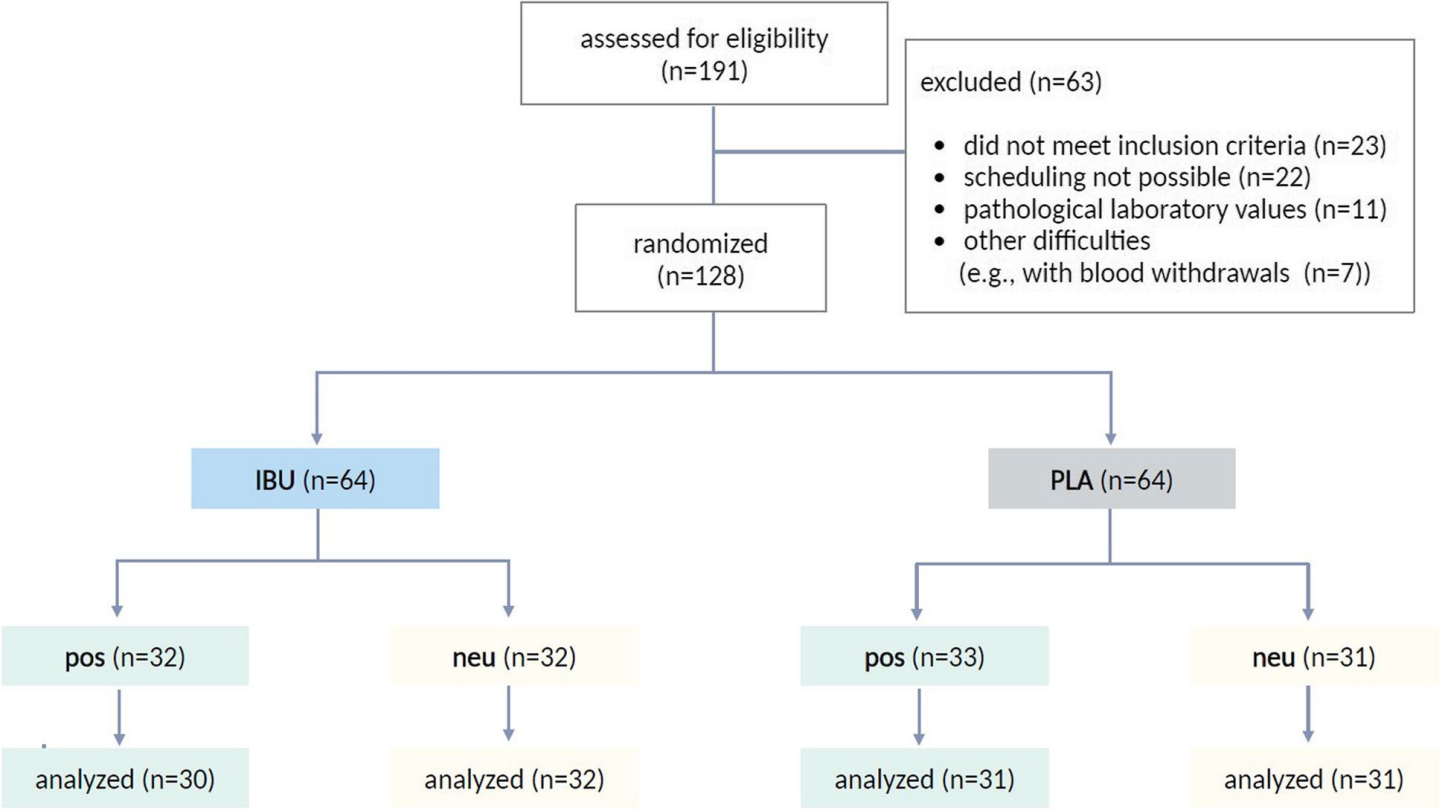
Flowchart illustrating the process of recruitment and data analysis. A total of *N* = 191 volunteers were assessed for eligibility with a standardized screening procedure. Of these, *N* = 128 were randomized to receive ibuprofen (IBU) or placebo (PLA) (1st randomization factor “medication”) combined with either positive (pos) or neutral (neu) verbal treatment information (2nd randomization factor “labeling”). Four volunteers needed to be excluded after data acquisition, resulting in a final data set of *N* = 124. Created with BioRender.com

A total of *N* = 191 healthy volunteers were screened, and *N* = 128 were enrolled and randomized into the four experimental groups (Fig. 3). Four participants were excluded after data acquisition: two due to vomiting that interfered with assessments and two for acute distress caused by family circumstances. Consequently, data from *N* = 124 participants are reported herein (sex assigned at birth: *N* = 51 (41.1%) male, *N* = 73 (58.9%) female; mean age: 24.8 ± 0.4; age range: 19–45 years). The majority of participants reported prior use of ibuprofen within the last 12 months (70.5%, *N* = 86). The groups did not differ significantly in any psychosocial or psychological characteristics (Table 1).

**Table 1:** Sociodemographic and psychological group characteristics in the four experimental groups

	**IBU + pos *(n* = 30)**	**IBU + neu (*n* = 32)**	**PLA + pos (*n* = 31)**	**PLA + neu (*n* = 31)**
Age (years)	24.5 ± 1.0	25.8 ±.9	24.9 ±.8	24.0 ±.6
% Women (*n*)	60 (18)	62.5 (20)	54.8 (17)	58.1 (18)
% Men (*n*)	40 (12)	37.5 (12)	45.2 (14)	41.9 (13)
BMI (kg/m^2^)	22.2 ±.4	23.2 ±.6	23.9 ±.6	22.6 ±.3
IBU use in past 12 months, % (n)*	60 (18)	59.4 (19)	75.9 (22)	87.1 (27)
Previous IBU treatment was effective * (% (*n*) of row above)	88.9 (16)	94.7 (18)	95.5 (21)	96.3 (26)
Education ≥ 12 years % (*n*)	96.7 (29)	93.8 (30)	93.5 (29)	93.5 (29)
STADI anxiety (trait version)	17.9 ± 1.0	17.3 ±.7	15.2 ±.7	16.5 ±.8
STADI depression (trait version)	15.9 ±.8	15.7 ±.7	14.5 ±.6	14.3 ±.7
STADI global (trait version)	33.8 ± 1.7	32.9 ± 1.3	29.7 ± 1.2	30.9 ± 1.3

Data are presented as means ± SEM or % (*n*). Groups were compared using univariate ANOVA or chi^2^-tests for dichotomous variables, with no statistically significant differences found (all *p* >.05)

*BMI* Body mass index, *STADI* State-Trait Anxiety Depression Inventory, *IBU* Ibuprofen, *PLA* Placeb, *Pos* Positive labeling, *Neu* Neutral labeling

*Values refer to proportions in each group who occasionally used over-the-counter ibuprofen within the last 12 months before study participation (regular use was exclusionary). Missing data for *n* = 2 participants within PLA + pos group; therefore, the percentages are based on the remaining *n* = 29 participants)

### Inflammatory, neuroendocrine and vital parameters

**Fig. 4:**
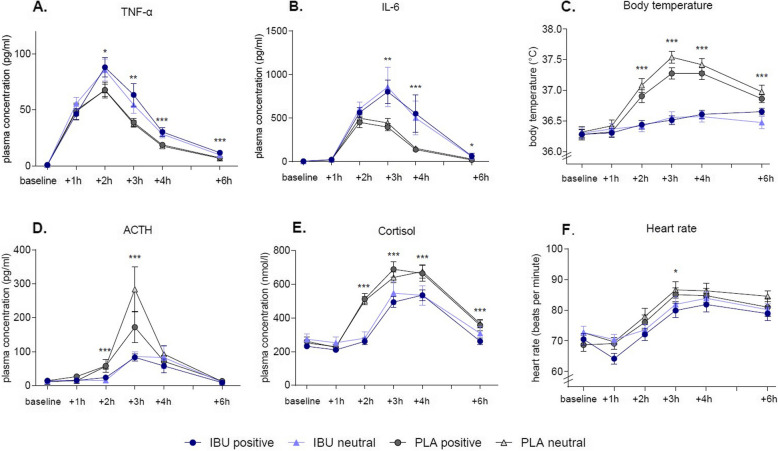
Physiological responses in the four experimental groups.  TNFα (**A**), Interleukin (IL)-6 (**B**), body temperature (**C**), ACTH (**D**), cortisol (**E**), and heart rate (**F**) were assessed at baseline and 1, 2, 3, 4, and 6 h post-injection. All parameters showed transient increases in response to LPS and were modulated by medication, but not by labeling; *p <.05, **p <.01, ***p <.001, IBU (ibuprofen) vs. PLA (placebo); results of post hoc *t*-tests. For ANOVA results, see text

Consistent with the characteristic LPS-induced acute systemic inflammatory response, transient increases in pro-inflammatory cytokines (ANOVA *time* effects for TNF-α: *F*_(1.3, 158.8)_ = 637.1; *p* < 0.001, *η*_*p*_^2^ = 0.84, Fig. 4A; IL-6: *F*_(2.8, 303.04)_ = 1009.3; *p* < 0.001, *η*_*p*_^2^ = 0.90, Fig. 4B) were accompanied by the typical changes in vital parameters (body temperature: *F*_(4.1, 485.6)_ = 115.8; *p* < 0.001, *η*_*p*_^2^ = 0.49, Fig. 4C; heart rate: *F*_(4.3, 517.1)_ = 104.9; *p* < 0.001, *η*^2^ = 0.47, Fig. 4F), observable in all experimental groups. Furthermore, plasma concentrations of ACTH (*F*_(3.2, 369.9)_ = 154.01; *p* < 0.001, *η*_*p*_^2^ = 0.57, Fig. 4D) and cortisol (*F*_(3.2, 369.9)_ = 178.6; *p* < 0.001, *η*_*p*_^2^ = 0.61, Fig. 4E) increased after LPS-administration, confirming activation of the HPA axis in response to systemic inflammation.

Testing the effects of ibuprofen vs. placebo treatment on markers of inflammation (based on ANOVA interaction effects of *time* × *medication*) revealed elevated plasma cytokine concentrations (TNF-α: *F*_(1.3, 158.8)_ = 4.9; *p* = 0.02, *η*_*p*_^2^ = 0.04; IL-6: *F*_(2.8, 303.0)_ = 4.9; *p* = 0.003, *η*_*p*_^2^ = 0.04). Ibuprofen further inhibited LPS-induced increases of body temperature (*F*_(4.1, 485.6)_ = 47.9; *p* < 0.001, *η*^2^ = 0.29) and heart rate (*F*_(4.3, 517.1)_ = 2.5; *p* = 0.03, *η*^2^ = 0.02), and lowered plasma concentrations of ACTH (*F*_(3.2, 369.9)_ = 9.3; *p* < 0.001, *η*_*p*_^2^ = 0.07) and cortisol (*F*_(3.2, 369.9)_ = 16.8; *p* < 0.001, *η*_*p*_^2^ = 0.13). Results of post hoc testing at individual time points are provided for all variables in Fig. 4. Hence, ibuprofen exerted expected effects on LPS-induced cytokine, vital, and neuroendocrine markers of inflammation, based on several previous endotoxin trials [28].

Labeling did not significantly affect any of these measures during experimentally induced systemic inflammation (all ANOVA *time* × *labeling* and *time* × *labeling* × *medication* interactions not significant, all *p* > 0.2).

### Bodily sickness symptoms

**Fig. 5:**
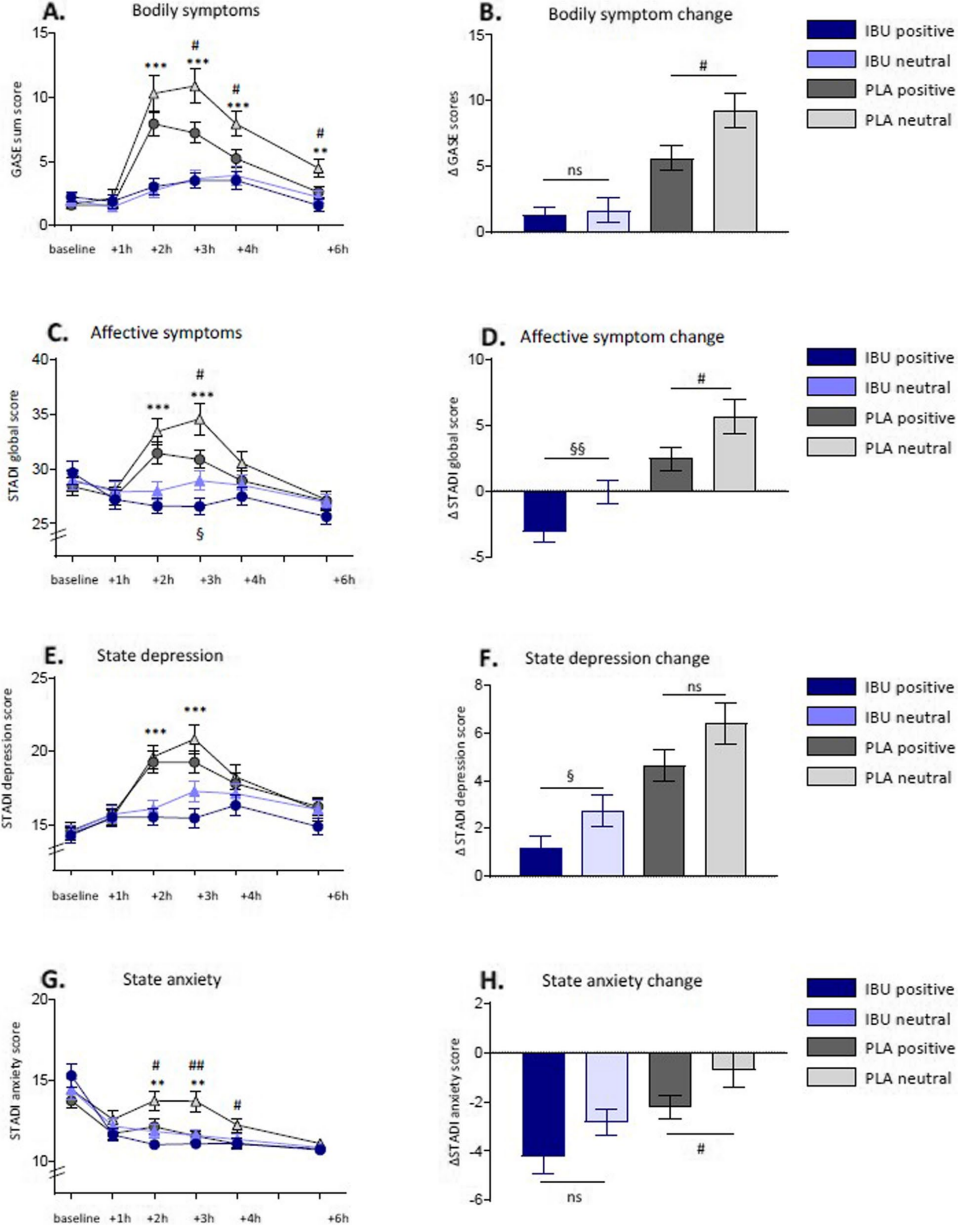
Self-reported sickness symptoms in the four experimental groups. Left panel: (**A**) Bodily sickness symptoms (GASE scores) and (**C**) affective symptoms (STADI global scores), comprised of (**E**) state depression (STADI subscale depression) and (**G**) state anxiety (STADI subscale anxiety) were repeatedly assessed at baseline and 1, 2, 3, 4, and 6 h after LPS injection. All parameters showed transient increases in response to LPS. All symptoms were reduced by ibuprofen compared to placebo (*p <.05, **p <.01, ***p <.001, IBUprofen vs. PLAcebo). Positive labeling reduced bodily and affective symptoms within the placebo groups (#*p* <.05, positive vs. neutral labeling) and reduced negative affectivity (STADI global score) within the ibuprofen groups (§*p* <.05, positive vs. neutral labeling); results of post hoc *t*-tests. For rm ANOVA results, see text. Right panel: Changes in GASE (**B**) and STADI (**D**, **F**, **H**) scores from baseline to the peak of inflammation (i.e., 3 h post-injection). Higher positive change scores indicate a greater increase in symptoms. Please note that change scores for STADI anxiety are negative due to increased (anticipatory) anxiety at baseline. §*p* <.05, §§*p* <.01, IBUprofen positive vs. IBUprofen neutral, #*p* <.05, PLAcebo positive vs. PLAcebo neutral. GASE, Generic Assessment of Side Effects Questionnaire; STADI, State-Trait Anxiety Depression Inventory

In all experimental groups, bodily symptoms were transiently increased after LPS administration (GASE score: *F*_(3.8, 450.3)_ = 51.6; *p* < 0.001, *η*_*p*_^2^ = 0.30; ANOVA *time* effect). Compared to placebo treatment, volunteers treated with ibuprofen reported significantly fewer symptoms (GASE score: *F*_(3.8, 450.3)_ = 22.4; *p* < 0.001, *η*_*p*_^2^ = 0.16; ANOVA *time* × *medication* interaction effect, see Fig. 5A for results of post hoc testing). Positive labeling further ameliorated bodily symptoms during experimental endotoxemia, as evidenced by a significant between-subjects *medication* × *labeling* interaction (GASE score: *F*_(1, 120)_ = 4.0; *p* = 0.05, *η*_*p*_^2^ = 0.03).

In secondary analyses, we tested labeling effects within the ibuprofen-treated and placebo-treated study arms separately. For bodily sickness symptoms, the mere expectation of receiving ibuprofen (i.e., positive treatment information in volunteers who in reality received placebo) in the placebo arm led to a significant improvement in bodily sickness symptoms compared to placebo-treated participants who received neutral treatment information. Within the ibuprofen arm, no effect of labeling was observable (for detailed results of post hoc testing see Fig. 5A and B).

Twenty-four hours after LPS application, volunteers retrospectively reported bodily symptoms experienced during the last 24 h. Less intense symptoms were reported in the positive when compared to the neutral labeling groups both within the ibuprofen arm (1.9 ± 0.6 versus 4.0 ± 0.9, *p* = 0.05) and the placebo arm (2.8 ± 0.8 versus 5.8 ± 1.0, *p* = 0.03) (results of independent t-tests).

### Affective symptoms

Negative affective symptoms were significantly increased after LPS administration in all experimental groups (STADI global score: *F*_(4.1, 494.1)_ = 19.6; *p* < 0.001, *η*_*p*_^2^ = 0.14; ANOVA *time* effect). Treatment with ibuprofen, compared to placebo, significantly reduced symptoms (STADI global score: *F*_(4.1, 494.1)_ = 16.9; *p* < 0.001, *η*_*p*_^2^ = 0.12; ANOVA *time* × *medication* interaction effect, see Fig. 5C for results of post hoc testing). Consistent with our hypothesis that positive labeling improves symptoms, affective symptoms were lower in volunteers of the positive labeling group when compared to controls, irrespective of medication condition (*F*_(4.1, 494.1)_ = 3.2; *p* = 0.01, *η*_*p*_^2^ = 0.03; ANOVA *time* × *labeling* interaction effect).

In secondary analyses, labeling effects within the ibuprofen-treated and placebo-treated study arms were tested separately. Analyses within placebo-treated volunteers revealed that positive labeling ameliorated symptoms, with expected ibuprofen treatment (placebo in reality) reducing symptoms (see Fig. 5C and D for results of post hoc testing). Analyses within the ibuprofen arms, i.e. among volunteers treated with ibuprofen, positive labeling further enhanced ibuprofen efficacy as these volunteers reported lower symptoms than volunteers in the neutral labeling control group (see Fig. 5C and D for results of post hoc testing).

To further elucidate changes in negative affective states, we analyzed the depression and anxiety subscales of the STADI questionnaire separately. These complementary analyses revealed a symptom-relieving effect of ibuprofen on state depression, as supported by a significant ANOVA *time* × *medication* interaction (STADI depression score: *F*_(4.3, 515.5)_ = 16.6; *p* < 0.001, *η*_*p*_^2^ = 0.12, see Fig. 5E and F for results of post hoc testing). State anxiety was also significantly reduced by ibuprofen treatment (*F*_(3.3, 390.5)_ = 6.4; *p* < 0.001, *η*_*p*_^2^ = 0.05; ANOVA *time* × *medication* interaction). Additionally, positive labeling independently reduced state anxiety (STADI anxiety score: *F*_(3.3, 390.5)_ = 2.8; *p* = 0.04, *η*_*p*_^2^ = 0.02; ANOVA *time* × *labeling* interaction, see Fig. 5G and H for results of post hoc testing).

### Additional measures of mood-related sickness symptoms and fatigue

Additional analyses of state anxiety (STAI state) and positive mood (MDBF) scores largely confirmed the results for STADI anxiety and depression scores. For LPS-induced fatigue (KSS), the analysis revealed a significant relieving effect of ibuprofen; however, there were no *labeling* or *labeling* × *medication* effects (see Additional file 2, section S1, Fig. S1). Data on affective and fatigue symptoms collected 24 h post-injection are reported in Additional file 2 (section S2, Table S1). See Additional file 2 (section S3, Table S2) for results of additional correlational and regression analyses on associations of affective symptoms with bodily symptoms and inflammatory markers.

## Discussion

We tested the hypotheses that the physician communication designed to enhance positive expectations regarding an anti-inflammatory treatment can alleviate sickness symptoms and enhance the efficacy of ibuprofen during endotoxemia. To this end, we conducted one of the largest endotoxin studies to date, capitalizing on LPS-induced endotoxemia as a translational model of inflammation. In a total sample of 124 healthy volunteers, LPS induced transient, well-characterized changes in immune, vital, and neuroendocrine markers of inflammation. Sickness-related bodily and affective symptoms were successfully elicited, replicating findings from previous LPS studies (e.g., [19, 20, 25, 32]). These symptoms were effectively alleviated by the dual COX1 and COX2 inhibitor ibuprofen, presumably via effects on the arachidonic acid and kynurenine metabolic pathways involved in affective symptoms [33]. This is noteworthy in light of the transdiagnostic significance of inflammation-induced sickness symptoms in chronic inflammatory conditions and psychiatric disorders [6, 7].

Positive labeling of ibuprofen treatment during the physician communication significantly alleviated both bodily and affective symptoms induced by acute inflammation, supporting our first hypothesis. Expanding on previous correlative evidence that expectations influence symptom experience during endotoxemia [17, 18], this study is the first to demonstrate the direct impact of positive physician communication. With the exception of fatigue, symptoms were alleviated by positive labeling even when an inert placebo rather than ibuprofen was administered. These findings in the placebo groups contained within our fully balanced design represent the typical study groups in placebo research, testing effects of placebo interventions in the context of inert substances. Complementing knowledge about placebo effects across health outcomes [4, 10], we herein provide novel proof-of-concept evidence that inflammation-induced sickness symptoms are modifiable by placebo.

Intriguingly, the response to *actual* ibuprofen treatment was also influenced by positive labeling. As hypothesized, affective symptoms were more effectively reduced when ibuprofen treatment was paired with positive labeling of the treatment. Analyses of pharmacological RCTs show that up to 70% of the total treatment effect is attributable to non-pharmacological effects, which include expectation effects [34]. Building on this evidence, a growing body of research highlights improved drug treatment outcomes through enhanced positive treatment expectations, particularly for analgesic drugs (e.g., [11–14]). In a notable clinical trial in migraine patients, a positive labeling of a treatment as rizatriptan boosted the efficacy of both placebo and medication treatment [14]. In our study, we demonstrated for the first time a similar “drug-boosting” effect on inflammation-induced negative affective states. On the other hand, no effect of labeling was observed on ibuprofen-induced relief of bodily symptoms, likely due to the drug's potent pharmacological action, which nearly eliminated symptom responses. It is conceivable that placebo effects might emerge with less effective medications or lower dosages [4]. A significant proportion of our study participants reported the occasional use of ibuprofen, with an overall positive treatment experience. Such previous experience with an effective treatment can shape current expectations, which may distinguish the present findings from studies with a newly introduced drug [14] or even with negative previous experience [35].

Negative affective symptoms, especially anxiety and depression, constitute a major source of suffering in patients with chronic inflammatory diseases, which often involve high rates of psychiatric comorbidity [36]. Systematically targeting positive treatment-related expectations by communication and other placebo strategies could alleviate emotional burden and resulting mental health impairments. Indeed, our data support that even small adjustments in health provider communication about treatments may improve self-reported treatment outcomes. Comparable effects of brief interventions, such as subtle changes in word choice [37], minor adjustments to therapy information [13], labeling [14], or framing of treatments [4], have been shown in other medical contexts, including vaccinations [38].

We explored the effects of our expectation intervention on physiological responses to LPS, including plasma cytokines and HPA-axis activity markers, but found no significant group differences. This aligns with the broad consensus that immune functions are generally unresponsive to verbal suggestion and are instead influenced by (unconscious) associative learning processes, often referred to as learned placebo effects [2]. Such effects have been observed, for example, in T-cell proliferation in kidney transplant patients [39]. Given that immune parameters were unaffected by labeling, mechanisms underlying the observed expectancy effects on self-reported outcomes are likely mediated by several biological systems, as suggested by placebo research. These include the endogenous opioid system, cannabinoid and dopaminergic pathways, and changes in brain network activity related to pain and emotion processing (for review, see [5]). These mechanisms represent potential pathways underlying the expectation effects observed in our study.

It also cannot be ruled out that positive labeling influenced immunobiological pathways underlying the symptoms induced by LPS. These pathways include cytokine-mediated activation of the afferent vagus nerve, which affects brain regions involved in interoception, and glial cell activation by proinflammatory cytokines and prostaglandins (for reviews, see [7, 15, 36]). Central prostaglandin synthesis, for example, appears to influence dopaminergic cells in the striatum and subsequently motivational processes associated with sickness symptoms during immune activation [40]. Evidence for expectancy effects on prostaglandin levels as mediators of nocebo effects comes from a study on altitude headache [41]. Participants who anticipated severe headaches at high altitudes experienced higher headache incidence and pain burden after ascending to 3500 m, which correlated with elevated prostaglandin E2 levels. Notably, the intake of a placebo pill without an active ingredient reduced both prostaglandin levels and headache severity. Clearly, mechanistic research is required to clarify the neurobiological mechanisms underlying placebo effects in inflammation-induced sickness. Irrespective of underlying mechanism(s), it should be noted that previous clinical studies in patients with chronic conditions such as asthma and chronic leg ulcers have also demonstrated a ‘disconnect’ between patient-reported outcomes and objective disease measures. Improvements in patient-reported outcomes, even in the absence of objective changes, are highly relevant for clinical practice. Such improvements are associated with better quality of life and increased therapy adherence [42].

Limitations of our work comprise the study sample consisting of healthy and young volunteers, which do not represent individuals at risk or patient cohorts. While we included men and women, the sample size was too small to address differences based on sex or gender, although this is called for given the female preponderance of many chronic inflammatory conditions as well as depression and anxiety. The findings were generated in an acute model of systemic inflammation. We chose the translational LPS model because it allows to induce clinically relevant symptoms and to assess symptom modulation under strictly standardized experimental conditions, which is not unconditionally possible in patients with inflammatory conditions and diverse treatment histories [35]. However, this may not reflect conditions involving recurring inflammatory bouts or chronic low-grade inflammation, calling for future work in real clinical settings. Further, the ibuprofen dose of 600 mg chosen herein virtually eliminated bodily symptoms and state anxiety. Thus, ibuprofen was unintendedly too effective, which may have overshadowed labeling effects. Finally, the neutral labeling condition comprised the information of a 50%/50% chance to receive ibuprofen, which is an established procedure in balanced placebo designs [4] and was chosen herein for ethical reasons, i.e., to avoid deceptive (blinded) drug application. However, while our approach may induce at least some positive expectations to receive a “real” drug, a hidden application of ibuprofen would likely have increased the difference to the positive labeling condition. Thus, our findings may rather be considered as conservative and underestimate the effect of labeling. This is of specific importance given that many patients are not aware or adequately informed about drug treatments, which is equivalent to a hidden treatment.

## Conclusions

In conclusion, this study provides the first experimental evidence in healthy individuals that positive labeling of a treatment, communicated within a psychosocial context, can independently reduce sickness symptoms and enhance the efficacy of ibuprofen in alleviating psychological distress during acute systemic inflammation. These findings extend prior work on placebo and labeling effects by demonstrating that expectations can boost the effectiveness of a well-established anti-inflammatory drug, particularly in alleviating affective symptoms. Notably, labeling did not alter physiological markers of inflammation, suggesting that the observed benefits are likely mediated by mechanisms distinct from direct immune modulation. Our results highlight the value of integrating psychological and pharmacological approaches to optimize treatment outcomes, especially those related to patient-reported symptoms of sickness behavior. These insights have practical implications for clinical settings, where positively framing treatments may serve as a feasible and ethical strategy to enhance therapeutic benefit. Future research should explore structured communication training for healthcare providers to support implementation, with attention to tailoring strategies to diverse patients with distinct expectations, treatment histories, and therefore individual communication needs.

## Supplementary Information

Additional file 1. Data file with raw data reported on herein.

Additional file 2: Supplementary methods and results. Section S1 with Fig. S1: Additional measures of mood-related sickness symptoms and fatigue. Section S2 with Table S1: Self-reported sickness symptoms assessed 24 h after LPS injection. Section S3 with Table S2: Exploratory correlation and regression analyses.

## Data Availability

All data analysed during this study are included in this published article, see Additional File S1. Study protocols can be requested by the corresponding author.

## Abbreviations

ACTH: Adrenocorticotropic hormone
ANOVA: Analysis of variance
CRC: Collaborative Research Center
CRP: C-reactive protein
DF: Degree of freedom
DRKS: German Clinical Trials Register
EDTA: Ethylenediaminetetraacetic acid
GASE: Generic Assessment of Side Effects Scale
HADS: Hospital Anxiety and Depression Scale
HPA: Hypothalamic–pituitary–adrenal
IBU: Ibuprofen
IL-6: Interleukin-6
KSS: Karolinska Sleepiness Scale
LPS: Lipopolysaccharide
MDBF: Multi-Dimensional Mood Questionnaire
MDD: Minimum detectable dose
Neu: Neutral labeling condition
PLA: Placebo
Pos: Positive labeling condition
RM: Repeated measures
SEM: Standard error of the mean
SPSS: Statistical Package for the Social Sciences
STADI: State-Trait Anxiety Depression Inventory
STAI: State-Trait Anxiety Inventory
TNF: Tumor necrosis factor
