# Supplementary material for: Placebo effects improve sickness symptoms and drug efficacy during systemic inflammation: a randomized controlled trial in human experimental endotoxemia
Source: BMC Med. 2025 Aug 4;23:455. doi: 10.1186/s12916-025-04292-8 (PMC12320331; doi:10.1186/s12916-025-04292-8)
Supplement: Supplementary file 2 — Additional file 2: Supplementary methods and results. Section S1 with Fig. S1: Additional measures of mood-related sickness symptoms and fatigue. Section S2 with Table S1: Self-reported sickness symptoms assessed 24 h after LPS injection. Section S3 with Table S2: Exploratory correlation and regression analyses. [file 12916_2025_4292_MOESM2_ESM.pdf]

ADDITIONAL FILE 2

Placebo effects improve sickness symptoms and drug efficacy during systemic inflammation: A randomized controlled trial in human experimental endotoxemia

Justine Schmidt[1,2], Johanna Reinold[1,3], Hana Rohn[3], Manfred Schedlowski[2,4], Harald Engler[2], Sigrid Elsenbruch[5,6], Sven Benson[1,2*]

Section S1: Additional measures of mood-related sickness symptoms and fatigue

State anxiety, positive mood, and fatigue were assessed with validated questionnaires at baseline and 1, 2, 3, 4, and 6 hours after LPS injection. For details regarding study design, measurement points, and statistical analysis, please see main manuscript.

State Anxiety

Methods: State anxiety was assessed with the state version of the State-Trait-Anxiety-Inventory (STAI) (29), as previously accomplished in LPS studies (e.g., 19, 20). Briefly, the STAI state is comprised of 20 Likert-scaled items. Sum scores range from 20 to 80, with higher scores indicting more pronounced states of anxiety.

Results: In the present study, LPS-application led to transient increases in STAI state anxiety ($F_{(3.7, 445.2)}$=24.8; p<.001, η_p^2=.17, ANOVA *time effect*), which is in line with previous reports on LPS-induced changes in STAI state scores (2, 3, 5). Increases in state anxiety were reduced by ibuprofen treatment ($F_{(3.7, 445.2)}$=13.8; p<.001, η_p^2=.10; ANOVA interaction effects of *time x medication*), and – as a trend – also by labeling ($F_{(3.7, 445.2)}$=x.x; p=.09, η_p^2=.02; ANOVA interaction effects of *time x labeling*), largely in line with the results for the STADI anxiety score. Results of post-hoc testing at individual time points are provided in Figure S1.

Positive mood

Methods: Positive (versus negative) mood was measured with the German Multidimensional Mood Questionnaire (MDBF) (30), subscale "GS". MDBF-GS scores can range from 3 – 12, with lower scores indicating decreases in positive mood. LPS effects on MDBF-GS scores have consistently been shown in previous reports (19, 20).

Results: In line with previous findings (19, 20), transient decreases in positive mood were observed after LPS administration in the present study ($F_{(3.8,\ 458.5)}$=55.2; p<.001, η_p^2=.32, ANOVA *time effect*). Decreases in MDBF positive mood scores were ameliorated by ibuprofen treatment ($F_{(3.8,\ 458.5)}$=17.3; p<.001, η_p^2=.13, ANOVA interaction effect of *time x medication)* and by positive labeling ($F_{(3.8,\ 458.5)}$=2.4; p=.049, η_p^2=.02, ANOVA interaction effect of *time x labeling*). For results of post-hoc testing at individual time points, see Figure S1.

Fatigue

Methods: Fatigue was assessed with the Karolinska Sleepiness Scale (KSS) (31). Participants are asked to choose one out of nine statements that best describes the present level of sleepiness or alertness, with scores ranging from 1 ("extremely awake") to 9 ("very sleepy, can only stay awake with great difficulty").

Results: In line with previous findings (32), transient decreases in KSS scores (indicating reduced wakefulness/increased sleepiness) were observed after LPS administration ($F_{(4.1,\ 489.1)}$=49.2; p<.001, η_p^2=.29, ANOVA *time effect*). Decreases in KSS scores were improved by ibuprofen treatment ($F_{(4.1,\ 489.1)}$=5.3; p<.001, η_p^2=.04, ANOVA interaction effect of *time x medication)*, but not by positive labeling ($F_{(4.1,\ 489.1)}$=0.3; p=.90, η_p^2=.003, ANOVA interaction effect of *time x labeling*). Results of post-hoc testing at individual time points are provided in Figure S1.

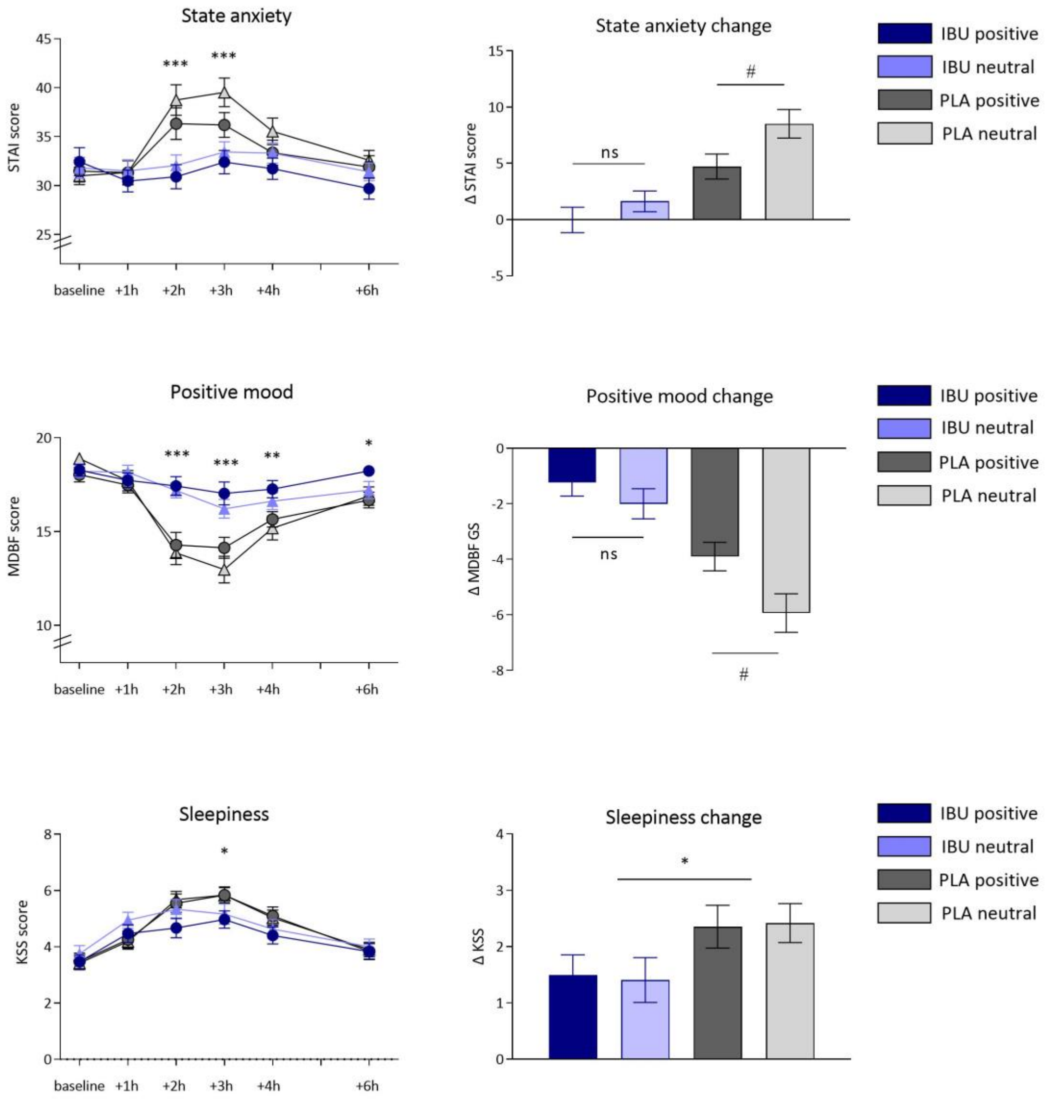

Figure S1: Self-reported state anxiety, mood, and fatigue in the four experimental groups. **Left panel:** State anxiety (STAI state scores, top), positive mood (MDBF scores, middle), and fatigue (KSS scores, bottom) were repeatedly assessed at baseline and 1, 2, 3, 4, and 6h after LPS injection. All parameters showed transient increases in response to LPS. All symptoms were reduced by ibuprofen compared to placebo (*p<.05, **p<.01, ***p<.001, IBUprofen vs. PLAcebo). For rm ANOVA results, see text. **Right panel:** Changes in STAI state (top), MDBF (middle) and KSS (bottom) scores from baseline to the peak of inflammation (i.e., 3h post-injection). For state anxiety and fatigue, higher positive changes scores indicate a greater increase in symptoms. For MDBF positive mood, greater negative change scores indicate a more pronounced decrease in positive mood. *p<.05, IBUprofen vs. PLAcebo. #p<.05, PLAcebo positive vs. PLAcebo neutral. STAI, State-Trait Anxiety Inventory; MDBF, Multidimensional Mood Questionnaire; KSS, Karolinska Sleepiness Scale.

<u>**Section S2: Self-reported sickness symptoms assessed 24 hours after LPS injection**</u>

Twenty-four hours after the LPS injection, volunteers were asked to rate acute affective symptoms (assessed with STADI state, STAI state, MDBF positive mood scales) and fatigue (KSS) (please see section S1 for information on questionnaires). Symptom scores were compared with univariate ANOVA. Significant differences were observed for positive mood (MDBF; $F_{(3,120)}$=2.9, p=.036) and fatigue (KSS, $F_{(3,120)}$=2.9, p=.035). State anxiety and depression symptoms were comparable between groups (Table S1).

Table S1: Self-reported sickness symptoms assessed 24h after LPS injection

Group<br>Scale	IBU+pos	IBU+neu	PLA+pos	PLA+neu
Affective symptoms (STADI global score)	24.5±.6	26.3±1.1	25.2±.7	24.5±.5
State anxiety (STADI anxiety score)	10.8±.2	11.3±.4	11.3±.4	11.0±.2
State depression (STADI depression score)	13.7±.6	14.9±.8	13.8±.5	13.5±.4
Positive mood (MDBF score)	19.2±.1*	17.8±.3*	18.8±.1	19.0±.1
State anxiety (STAI score)	28.7±.5	30.7±.7	28.4±.4	28.1±.4
Fatigue (KSS score)	2.5±.1	3.3±.1	2.3±.1	2.6±.1

Symptom scores, assessed twenty-four hours after the LPS injection. Data are shown as mean ± SEM. *p<.05, post-hoc comparisons within medication / placebo arms (one-tailed independent t-tests, Bonferroni-corrected for m=2 group comparisons). IBU+pos = ibuprofen + positive labeling, IBU+neu = ibuprofen + neutral labeling, PLA+pos = placebo + positive labeling, PLA+neu = placebo + neutral labeling

Section S3: Exploratory correlation and regression analyses

Methods: Exploratory correlation and regression analyses were computed within the full sample to examine whether inflammation-induced affective symptoms were associated with inflammatory markers and / or bodily symptoms. Correlations between changes in STADI global scores (as indicator of inflammation-induced affective symptoms), changes in bodily sickness symptoms (assessed with the Generic Assessment of Side Effects questionnaire, GASE), and changes in markers of systemic inflammation (TNF-α, IL-6, cortisol) were computed as Pearson's r. Change Scores were computed as delta (Δ) between peak value (i.e. 2h post-injection for TNF-α, 3h post-injection for all other parameters) and baseline value. To further analyze the interrelationship of parameters, a stepwise multiple linear regression analysis was conducted with Δ STADI global score as criterion, and Δ GASE score, Δ TNF-α, Δ IL-6, and Δ cortisol as predictor variables.

Results: Correlation analyses indicated significant associations of changes in STADI global scores with changes in plasma cortisol concentrations as well as with GASE scores (Table S2). Multiple regression analysis revealed that changes in STADI scores were significantly predicted by GASE score changes, but not by changes in cortisol concentrations ($F=149.1$, $p<.001$). GASE change scores predicted approximately 57% of the variance in STADI change scores ($B=0.753$, $\beta=0.759$; $t=12.21$, $p<.001$; adjusted $R^2=0.57$), while no additional variance was explained by changes in cortisol or cytokine concentrations. This suggests that the intensity of affective symptoms may rather be driven by the number and intensity of bodily symptoms, in line with the notion the sickness symptoms during systemic inflammation are rather not linearly associated inflammatory markers (8, 9)

Table S2: Correlations between changes in affective symptoms, bodily symptoms, and inflammatory markers

	ΔSTADI Global score	ΔSTADI depression	ΔSTADI anxiety	ΔGASE
ΔSTADI Global score	./.	**r=.84, p<.001**	**r=.76, p<.001**	**r=.76, p<.001**
ΔGASE	**r=.76, p<.001**	**r=.72, p<.001**	**r=.48, p<.001**	./.
ΔTNF-α	r=.04, p=.66	r=.05, p=.60	r=.01, p=.88	r=.03, p=.72
ΔIL-6	r=.01, p=.89	r=.06, p=.56	r=-.04, p=.66	r=-.02, p=.86
ΔCortisol	**r=.23, p=.01**	**r=.20, p=.024**	r=.17, p=.06	**r=.25, p=.005**

Results are were computed as Pearson's r within the full sample. Change scores (Δ) were computed as delta between peak (at 2 hours post-injection for TNF-α, 3 hours for all other parameters) minus baseline. Results are shown as r and *p*-values (two-tailed). STADI = State-Trait Anxiety and Depression Inventory, state version. GASE = Generic Assessment of Side Effects.
